# Induction of Autophagy as a Therapeutic Breakthrough for NAFLD: Current Evidence and Perspectives

**DOI:** 10.3390/biology14080989

**Published:** 2025-08-04

**Authors:** Yanke Liu, Mingkang Zhang, Yazhi Wang

**Affiliations:** 1 Department of Laboratory Medicine, The First Hospital of Lanzhou University, Lanzhou University, Lanzhou 730000, China; 2 School of Pharmacy, Lanzhou University, Lanzhou 730000, China; 3 The Second School of Clinical Medicine, Lanzhou University, Lanzhou 730000, China

**Keywords:** nonalcoholic fatty liver disease, autophagy, diet and exercise, pharmacological therapies

## Abstract

Nonalcoholic fatty liver disease (NAFLD) is a clinicopathological syndrome characterised by hepatic steatosis in the absence of significant alcohol consumption or other specific causes of liver injury. It has become one of the leading causes of liver dysfunction worldwide. However, the precise pathophysiological mechanisms underlying NAFLD remain unclear, and effective therapeutic strategies are still under investigation. Autophagy, a vital intracellular process in eukaryotic cells, enables the degradation and recycling of cytoplasmic components through a membrane trafficking pathway. Recent studies have demonstrated a strong association between impaired or deficient autophagy and the development and progression of NAFLD. Restoring autophagic function may represent a key approach to mitigating hepatocellular injury. Nevertheless, due to the complexity of autophagy regulation and its context-dependent effects on cellular function, therapeutic strategies targeting autophagy in NAFLD remain limited. This review aims to summarise the relationship between autophagy and NAFLD, focusing on autophagy as a central mechanism. We discuss the latest research advances regarding interventions such as diet and exercise, pharmacological therapies (including modern pharmacological therapy and plant-derived compounds), and other approaches (such as hormones, nanoparticles, gut microbiota, and vitamins). Furthermore, we briefly highlight potential autophagy-related molecular targets that may offer novel therapeutic insights for NAFLD management.

## Simple Summary

Nonalcoholic fatty liver disease (NAFLD) is characterized by hepatic steatosis occurring without significant alcohol consumption or other specific liver injury factors. The exact pathophysiological mechanisms underlying NAFLD remain incompletely understood. Autophagy is an intracellular process in eukaryotic cells involving the degradation and recycling of cytoplasmic components via membrane trafficking pathways. Impaired or defective autophagy is closely associated with the development and progression of NAFLD. Restoring autophagic function may represent a key pathway for alleviating hepatocyte injury. This review aims to summarize the association between autophagy and NAFLD, with a specific focus on the role of autophagy as a core mechanism. Recent research advances in dietary and exercise interventions, pharmacological treatments (including modern drug therapies and plant-derived compounds), and other approaches (such as hormones, nanoparticles, gut microbiota, and vitamins) are discussed. Additionally, a brief overview of autophagy-related molecular targets relevant to NAFLD treatment is provided.

## 1. Introduction

**Figure 1:**
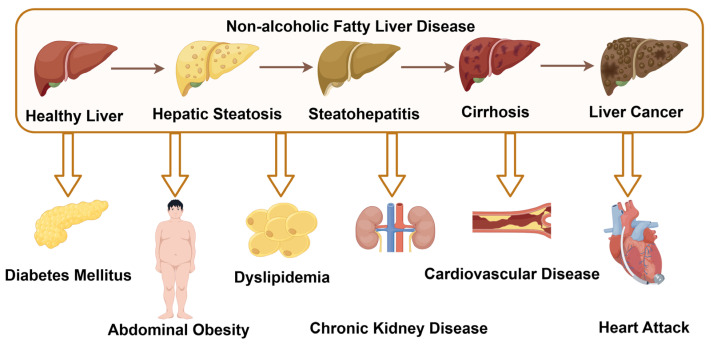
The progression of NAFLD and its associated complications (by Figdraw 2.0, ID = YRPUYadda8).

Nonalcoholic fatty liver disease (NAFLD) is a collective term for liver disorders associated with metabolic dysfunction, and it represents one of the most prevalent chronic liver diseases worldwide, affecting nearly 25% of the adult population. This imposes a significant burden on global healthcare systems. In the absence of excessive alcohol consumption or other liver diseases, NAFLD may progress from simple hepatic steatosis to nonalcoholic steatohepatitis (NASH), with or without hepatic fibrosis, and eventually to cirrhosis and hepatocellular carcinoma [1]. Moreover, as a multisystem disease, NAFLD markedly increases the risk of developing type 2 diabetes, central obesity, dyslipidemia, chronic kidney disease, cardiovascular diseases, and heart failure [2] (Figure 1). Unfortunately, due to its complex pathogenesis, no approved targeted therapies are currently available for NAFLD.

Autophagy is a vital process by which eukaryotic cells maintain intracellular homeostasis under stress conditions such as nutrient deprivation, infection, or hypoxia, through the clearance of damaged organelles, proteins, or cellular debris. Based on the pathway through which cellular components are delivered to lysosomes, autophagy is categorised into three main types: macroautophagy (which is the focus of this manuscript), microautophagy, and chaperone-mediated autophagy [3]. Among them, macroautophagy is the most extensively studied and commonly observed form. Briefly, intracellular substrates destined for degradation are sequestered by a double-membraned structure known as the isolation membrane, forming an autophagosome, which subsequently fuses with the lysosome to form an autolysosome where degradation occurs [3]. Autophagy can also be classified based on the nature of its cargo into selective and non-selective autophagy. Non-selective autophagy is typically activated under general stress conditions such as nutrient deprivation, during which it non-specifically engulfs cytoplasmic constituents (including organelles and proteins) for degradation and recycling into energy and metabolic intermediates. In contrast, selective autophagy specifically identifies and removes particular substrates, such as damaged mitochondria (termed mitophagy), lipid droplets (lipophagy) or portions of the endoplasmic reticulum (ER-phagy) [4].

Previous studies have demonstrated that autophagy is implicated in the pathogenesis of various conditions, including neurodegeneration, cancer, myopathies, infections, inflammatory diseases, and lysosomal storage disorders [5]. More recently, increasing evidence has revealed a close relationship between autophagy and NAFLD. Autophagy contributes to hepatic homeostasis by regulating lipid metabolism, improving insulin resistance (IR) and hepatocellular injury, and suppressing inflammation and endoplasmic reticulum stress (ERS) [6]. Clinical studies have shown that autophagosomes are markedly reduced and autophagic activity is impaired in liver biopsy specimens from patients with NAFLD [7]. Furthermore, impaired or defective autophagy may exacerbate hepatic lipid accumulation and worsen steatosis [8]. These findings suggest that restoration or induction of autophagy could represent a promising therapeutic strategy for the treatment of NAFLD.

## 2. Autophagy

Autophagy is a multistep process orchestrated by a group of evolutionarily conserved autophagy-related genes (ATGs). To date, more than 40 highly homologous ATGs have been identified in both yeast and mammalian cells. Under the regulation of these ATGs, the autophagic process proceeds in a well-coordinated manner. Autophagy involves several key steps: initiation and nucleation of the phagophore, elongation and expansion of the autophagosomal membrane, fusion of the autophagosome with the lysosome, and subsequent degradation and recycling of the sequestered intracellular contents within the autolysosome [9,10].

Under stress conditions such as nutrient deprivation, hypoxia, oxidative stress, infection, inflammation, or exposure to various chemical agents, the ULK1 complex composed of unc-51-like kinases 1 and 2 (ULK1 and ULK2), ATG13, ATG101, and focal adhesion kinase family interacting protein of 200 kDa (FIP200) becomes activated [9]. This complex phosphorylates the class III phosphatidylinositol 3-kinase (PI3K) complex, also known as the Beclin1–Vps34 complex, which includes ATG14, VPS15, VPS34, AMBRA1, UVRAG, and Beclin1. This phosphorylation event initiates the formation of the phagophore, a double-membraned isolation membrane that marks the beginning of autophagosome assembly.

**Figure 2:**
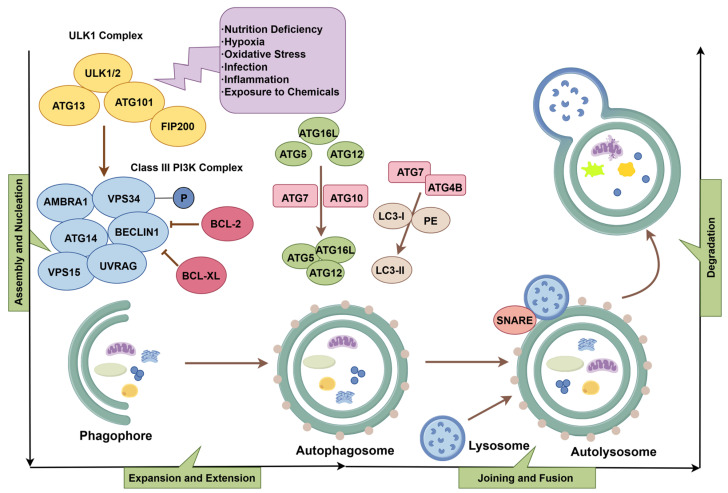
The process of autophagy (by Figdraw 2.0, ID = UTARU504f4).

Beclin1 can interact with anti-apoptotic proteins such as BCL-2 and BCL-XL, which inhibit autophagosome formation by suppressing Beclin1 activity. Subsequently, the E1- and E2-like enzymatic activities of ATG7 and ATG10 catalyse the conjugation of ATG5 to ATG12. The resulting complex interacts with ATG16L to form the ATG5–ATG12–ATG16L conjugate. At the same time, ATG7 promotes the processing of LC3 (microtubule-associated protein 1A/1B-light chain 3) via ATG4B, enabling the conjugation of LC3-I with phosphatidyl-ethanolamine (PE) to produce LC3-II, the membrane-bound form of LC3 [3]. These two conjugation systems cooperatively drive the elongation of the autophagosomal membrane. The fusion of the autophagosome with the lysosome is mediated by SNARE proteins such as STX17 and VAMP8. This fusion results in the formation of the autolysosome, where the sequestered intracellular contents are degraded by lysosomal hydrolases. The degradation products, including amino acids and fatty acids, can be recycled by the cell for reuse or further participation in metabolic pathways [11] (Figure 2).

## 3. Impaired Autophagy Aggravates NAFLD

**Figure 3:**
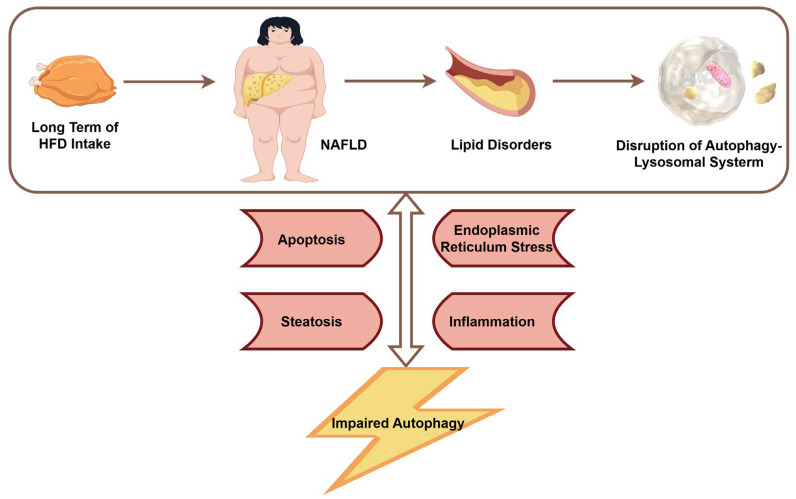
The relationship between NAFLD and autophagy (by Figdraw 2.0, ID = ITTRP111a0).

Under physiological conditions, autophagy plays a crucial role in maintaining hepatic metabolic homeostasis. During nutrient deprivation, hepatic autophagy is activated to promote the breakdown of intracellular substrates, thereby supplying energy to cells and organs. Conversely, under nutrient-rich conditions, autophagy is suppressed, favoring anabolic metabolism over catabolism [6]. Autophagy also alleviates hepatocellular damage induced by high-glucose and high-lipid conditions to some extent [12,13,14]. However, when autophagy is impaired or deficient, excessive accumulation of lipid droplets, inflammation, ERS, and apoptosis in the liver can aggravate metabolic dysregulation and contribute to the onset and progression of NAFLD [8] (Figure 3). For example, liver biopsy specimens from NAFLD patients have shown an association between hepatic inflammation and defective autophagic activity [15]. Another study indicated that impaired autophagic flux during NAFLD progression is related to ERS-induced hepatocyte apoptosis [16]. These findings suggest that inflammation and ERS may partly mediate autophagy dysfunction in NAFLD. Additional studies have confirmed that mice with autophagy deficiency exhibit hepatomegaly and parenchymal liver injury, characterised by inflammatory infiltration, hepatocyte apoptosis, pericellular fibrosis, and prominent proliferation of bile duct epithelial cells [17]. In patients with NASH, autophagy deficiency in liver sinusoidal endothelial cells has been shown to promote hepatic inflammation, endothelial-to-mesenchymal transition, apoptosis, and the development of liver fibrosis [18].

## 4. Restoration of Autophagy Ameliorates NAFLD

Based on the above findings, it can be inferred that autophagy dysfunction plays a critical role in the pathogenesis of NAFLD. Autophagy may represent an integrative therapeutic target for NAFLD, and restoring or inducing autophagic activity may attenuate or even halt disease progression. This provides a novel direction for the development of treatment strategies against NAFLD.

**Figure 4:**
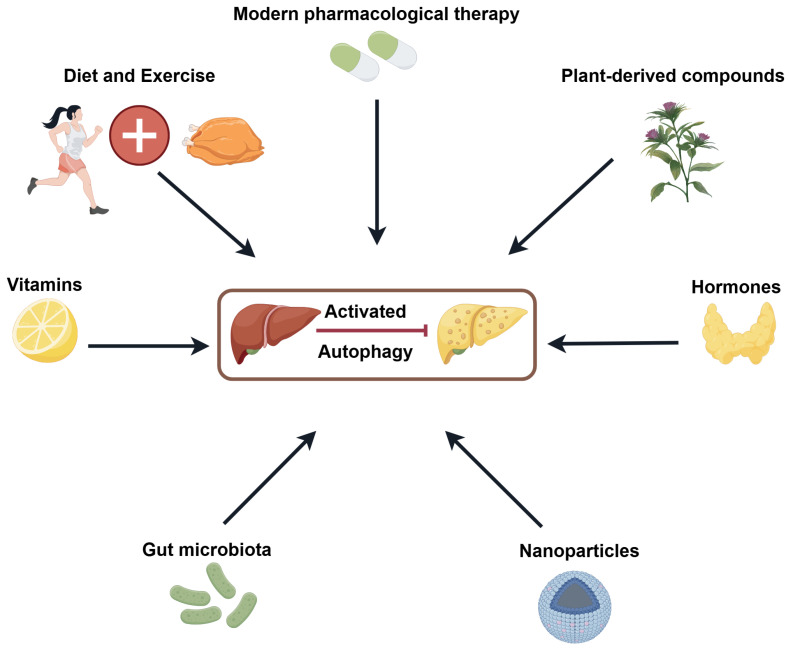
Strategies for treating NAFLD by inducing autophagy (by Figdraw 2.0, ID = PRUTU5c4a3).

In this context, we summarise recent therapeutic approaches aimed at enhancing autophagy, including lifestyle interventions such as diet and exercise, pharmacological agents including modern drugs and plant-derived compounds, and other interventions such as hormones, nanoparticles, gut microbiota modulation, and vitamins (Figure 4).

### 4.1. Diet and Exercise

A healthy diet combined with regular physical activity has long been regarded as the first-line therapeutic approach for NAFLD. Properly structured lifestyle interventions can help reduce body weight, alleviate hepatic inflammation, steatosis, and lipid accumulation, enhance liver function, and ultimately slow disease progression [19]. Mechanistic studies on the preventive and therapeutic effects of diet and exercise in NAFLD have been extensively conducted. These investigations primarily focus on improving hepatic lipid droplet dynamics, reducing inflammation, ERS, and oxidative stress, as well as restoring autophagy, mitochondrial dysfunction, and gut microbiota imbalance [20,21,22]. Among these mechanisms, the restoration of autophagy is considered one of the key contributors to the beneficial effects of dietary and exercise interventions in NAFLD management.

Previous studies have shown that chronic nutrient excess can induce hepatic steatosis and liver injury, potentially through mechanisms involving impaired autophagic activity and ERS [23]. In contrast, caloric restriction enhances hepatic autophagy, mitochondrial biogenesis, and the expression of inflammation-related proteins [24]. Intermittent fasting (IF) is a dietary strategy characterised by alternating periods of fasting and feeding, including protocols such as alternate-day fasting (ADF), time-restricted feeding (TRF), and the fasting-mimicking diet (FMD) [25]. IF can reduce the risk of metabolic disorders in overweight or obese individuals by alleviating oxidative stress, optimising circadian rhythm, and promoting ketogenesis. Strong evidence supports the therapeutic potential of IF in managing NAFLD [26]. Animal experiments have demonstrated that IF can significantly reduce body weight, liver weight, and the homeostasis model assessment of insulin resistance (HOMA-IR) index in NAFLD mouse models [27]. In addition, IF effectively decreases hepatic lipid accumulation and inflammation, thereby attenuating lipotoxicity associated with NAFLD [28,29]. Mechanistic studies have further revealed that IF exerts its protective effects by activating the MIF/AMPK and AMPK/ULK1 signalling pathways, and by inhibiting mTOR phosphorylation, thus regulating autophagy and apoptosis to improve hepatic function in NAFLD [27,29]. Beyond caloric restriction, several dietary supplements have shown promise in alleviating NAFLD by promoting hepatocellular autophagy. These include medium-chain fatty acids [30], corn peptides [31], and γ-linolenic acid [32], all of which reduce hepatic lipid accumulation. Notably, branched-chain amino acids (BCAAs) may activate the mTOR pathway, suppressing the conversion of free fatty acids (FFA) to triglycerides (TG) and inhibiting autophagy, thereby exacerbating hepatic lipotoxicity [33]. However, some studies suggest that moderate BCAA supplementation does not necessarily exacerbate insulin resistance, impair glucose tolerance, or directly induce lipotoxicity [34,35,36]. The observed inconsistencies across studies may be attributed to tissue-specific effects of BCAAs and variations in dietary contexts, which warrant further investigation for conclusive evidence.

In addition to dietary adjustment, exercise can also reduce hepatic lipid accumulation and improve NAFLD by enhancing hepatic autophagy [37,38]. Lysosomes are important organelles for degrading intracytoplasmic lipid droplets (LDs). Lipophagy is a type of selective autophagy that targets lipid droplets for degradation to maintain cellular lipid homeostasis [39]. Exercise can promote lipophagy via regulating lysosome number and function, which further ameliorates hepatic steatosis [40]. Obesity, hepatic steatosis, inflammation and hepatic injury significantly improved in NAFLD mice after 15 weeks of aerobic plate training (60 min/day, 5 days/week). Further studies revealed that exercise regulated hepatic LDs dynamics by inhibiting the expansion of abnormal LDs, promoting lysosomal co-localisation with LDs during lipid phagocytosis, and inducing lysosomal clearance of LDs [41]. Similarly, ET (endurance training) and VPA (voluntary physical activity) improved hepatic mitochondrial biogenesis-related proteins and autophagy signalling. In addition, ET reduced susceptibility to hepatic mitochondrial permeability transition pore (mPTP) and positively regulated factors associated with mitochondrial transcription, fusion and autophagy. It hinted that autophagy/mitochondrial autophagy induction may be an important approach for exercise to protect the liver [42]. In addition, certain proteins that regulate lipids may also serve as important bridges that link motility and autophagy. Fatty acid-binding protein (FABP1) is a hepatic fatty acid binding protein that inhibits TG metabolism, cholesterol uptake and lipid transport [43]. A research team trained NAFLD mice to swim for 12 weeks and discovered that exercise down-regulated FABP1, which subsequently restored lysosomal protease activity and lysosomal acidification, significantly increased autophagic flux, and preserved lipid homeostasis in the liver [44]. In addition, exercise attenuates hepatic steatosis by activating autophagy through AMPK-related pathways. Guarino et al. [45] demonstrated that exercise increased LC3-II/LC3-I and activated the AMPK/mTOR pathway during improving biochemical and histological parameters in NAFLD. Furthermore, Li et al. [46] showed that exercise also ameliorated LDs metabolic disorders in NAFLD by activating the AMPK/Sirtuin1 (SIRT1) pathway and lipophagy. Recently, studies that adopted a combination of diet and exercise strategies have also found that hepatic autophagy played an important role in slowing down the process of NAFLD. The specific mechanism was related to the reduction of inflammation and ERS, activation of the AMPK/ULK1 pathway, and inhibition of the Akt/mTOR/ULK1 pathway [47,48]. Notably, current evidence demonstrates a dose-response relationship between exercise and autophagy activation. Moderate-to-high intensity aerobic exercise with intermediate duration (e.g., 30–60 min at 50–70% VO_2_max) appears most effective in inducing physiological autophagy [49,50]. However, optimal dosing should be individualized based on factors such as age, health status, and environmental conditions [51,52]. Taken together, the above studies suggested that regulation of diet and/or exercise-related lifestyle can help delay the progression of NAFLD, and one important mechanism may be related to the activation of autophagy (Table 1).

### 4.2. Modern Pharmacological Therapy

For metabolic diseases such as NAFLD, pharmacological intervention should be considered when lifestyle management alone fails to control disease progression. The development and progression of NAFLD are closely associated with lipotoxicity, insulin resistance (IR), oxidative stress, and inflammation. Therefore, drugs that regulate glucose and lipid metabolism, improve IR, and possess anti-inflammatory and antioxidant properties may be beneficial for the treatment of NAFLD. In recent years, emerging evidence has demonstrated that modern pharmacological therapies, including glucose-lowering and lipid-lowering agents, can effectively reduce hepatic lipid accumulation and alleviate liver fibrosis. Notably, these therapeutic effects are, at least in part, mediated through the restoration of autophagic homeostasis.

#### 4.2.1. SGLT-2i

Sodium-glucose cotransporter 2 inhibitors (SGLT2i) are a new class of oral hypoglycemic agents. At present, several SGLT2 inhibitors, including dapagliflozin, canagliflozin, empagliflozin, and ertugliflozin, have been approved by the United States Food and Drug Administration [53]. These drugs exert their hypoglycemic effects primarily by inhibiting sodium-glucose cotransporters in the renal proximal tubules, thereby preventing glucose reabsorption. In addition to lowering blood glucose levels in patients with diabetes, they have also been shown to protect the kidney and cardiovascular system, reduce visceral and ectopic fat, improve lipid profiles and insulin resistance, and lower body weight, serum uric acid, and blood pressure [54]. In recent years, increasing evidence has demonstrated the potential of SGLT2 inhibitors as promising therapeutic agents for the treatment of NAFLD. The underlying mechanisms may be closely related to the induction of autophagy [55]. For example, empagliflozin enhances autophagy in hepatic macrophages through the AMPK and mTOR signalling pathways. This activation further suppresses inflammation mediated by the interleukin 17 and interleukin 23 axis, thereby reducing liver injury in mouse models of NAFLD combined with type 2 diabetes mellitus [56]. In a recent study, Chun and colleagues reported that SGLT2 expression is elevated in the liver tissues of patients with nonalcoholic steatohepatitis. This finding provides a theoretical basis for the hepatic action of SGLT2 inhibitors [57]. Empagliflozin has also been found to activate the AMPK and TFEB pathway by reducing O-GlcNAcylation levels in the liver. This leads to enhanced autophagic flux and ultimately attenuates hepatic lipid accumulation, inflammation, and fibrosis [57]. Recent studies have also shown that dapagliflozin and canagliflozin improve NAFLD through autophagy regulation [58,59]. Dapagliflozin increases the levels of autophagy-related markers such as LC3B and Beclin1, reduces p62 expression, and induces autophagy through the AMPK and mTOR pathway [59]. Canagliflozin promotes autophagy by increasing the ratio of LC3 II to LC3 I and by upregulating Atg7, thereby regulating hepatic lipid metabolism and suppressing inflammation [58]. Taken together, SGLT2 inhibitors may alleviate hepatic steatosis by activating autophagy. They represent a potentially effective therapeutic approach for NAFLD and offer new perspectives for clinical application.

#### 4.2.2. GLP1-RA

Glucagon-like peptide 1 (GLP-1) is an incretin hormone that stimulates insulin secretion in a glucose-dependent manner upon binding to its receptor. It also inhibits glucagon secretion and exerts additional effects, including anti-inflammatory activity, cardiovascular protection, delayed gastric emptying, and regulation of lipid metabolism. Currently, GLP-1 receptor agonists (GLP-1RAs) are widely used in the clinical management of diabetes and obesity [60,61]. Recent studies have demonstrated that GLP-1RAs can improve liver injury and metabolic disturbances in patients with NAFLD. Similar to the mechanism of action of sodium-glucose cotransporter 2 inhibitors, GLP-1RAs improve NAFLD by suppressing hepatic inflammation and oxidative stress, enhancing lipid metabolism, and promoting autophagy [62]. For instance, liraglutide has been shown to induce autophagy through the SIRT1 and SIRT3-mediated FOXO3a and LC3 pathway, and to enhance mitochondrial structure by upregulating proteins involved in mitochondrial fission, fusion, and the respiratory chain. These effects ultimately reduce oxidative stress and improve hepatic function in NAFLD [63]. Furthermore, liraglutide can induce the expression of autophagy-related proteins such as LC3B, Beclin1, and Atg7, and activate both the AMPK and mTOR signalling pathway and the ROR alpha-mediated autophagy pathway, thereby reducing hepatic lipid deposition. Similarly, Yu and colleagues found that liraglutide alleviates mitochondrial dysfunction and reactive oxygen species generation in nonalcoholic steatohepatitis by promoting mitophagy [64]. Exenatide has also been reported to reduce oxidative stress and inhibit the NLRP3 inflammasome by enhancing hepatic autophagy and mitophagy. These effects contribute to the protection of liver function in mice with NAFLD and type 2 diabetes mellitus [65]. Moreover, exenatide can activate the AKT and mTOR pathway and promote the autophagy lysosome pathway, thereby increasing autophagic flux and reducing lipotoxicity and lipid accumulation in hepatocytes [66,67]. These findings suggest that GLP-1 receptor agonists may represent an important therapeutic strategy against NAFLD by promoting hepatic autophagy, regulating mitochondrial function and structure, and suppressing inflammation and oxidative stress. In the future, GLP-1RAs may become a valuable treatment option for patients with NAFLD. Table S1 provides a comprehensive comparison of the drug profiles between SGLT-2i and GLP-1RAs.

#### 4.2.3. Biguanides

Metformin is the principal member of the biguanide class of glucose-lowering agents. It suppresses hepatic gluconeogenesis, enhances fatty acid oxidation, inhibits lipogenesis, and increases insulin sensitivity. Recent studies have applied metformin to the management of nonalcoholic fatty liver disease and have shown that it can improve the condition by inducing autophagy [68]. Metformin activates autophagy by down-regulating STAT3 and simultaneously reduces the expression of inflammatory cytokines such as IL-1β, IL-6, and TNF-α, thereby exerting therapeutic effects in nonalcoholic steatohepatitis [69]. Song and colleagues [70] further demonstrated that metformin alleviates hepatocellular lipid accumulation by stimulating SIRT1-mediated autophagy through an AMPK-independent mechanism. It can also promote Parkin-mediated mitophagy, improving hepatic lipid metabolism [71]. Zhang et al. [72] confirmed that metformin attenuates hepatic steatosis and insulin resistance in mouse models of nonalcoholic fatty liver disease by enhancing TFEB-mediated autophagy. The most recent research indicates that metformin activates tristetraprolin via the AMPK and SIRT1 pathway; tristetraprolin then suppresses inflammation to reduce hepatocellular necrosis and promotes hepatic lipophagy by inhibiting mTORC1 and increasing TFEB nuclear translocation [73]. Collectively, these findings suggest that metformin mitigates nonalcoholic fatty liver disease by activating hepatocellular autophagy through the modulation of multiple transcription factors.

#### 4.2.4. Lipid-Modifying Drugs

Under normal physiological conditions, triglycerides (TG) stored in LDs can be hydrolysed into free fatty acids (FFA) to supply energy to the body. In patients with NAFLD, the degradation of TG in LDs is impaired, leading to hepatic TG accumulation and the development of insulin resistance (IR). IR, in turn, promotes the influx of additional FFAs into the liver, further exacerbating hepatic lipid accumulation and steatosis [74]. Several lipid-lowering agents have been shown to delay the progression of NAFLD by stabilising lipid profiles, reducing IR, and alleviating oxidative stress and inflammation. Some studies suggest that the induction of autophagy may represent a key mechanism by which these lipid-modulating drugs exert their therapeutic effects on NAFLD. For instance, Yoo and colleagues [75] reported that fenofibrate reduces hepatic lipid accumulation by activating lipophagy and the transcription factors TFEB and TFE3 through PPARα agonism. Another recent study also confirmed that the beneficial effects of fenofibrate on hepatic steatosis, IR, and gut microbiota modulation depend on TFEB-mediated autophagy [76]. Ezetimibe has also been shown to alleviate hepatic steatosis and IR in obese and type 2 diabetic rats by inducing autophagy, which results in reduced serum glucose, insulin, and lipid levels [77]. Its mechanism involves the activation of the Nrf2-Keap1 antioxidant signalling pathway through p62-dependent autophagy, thereby protecting hepatocytes from oxidative injury [78]. In addition to these agents, other drugs from various therapeutic classes have also been reported to improve hepatic steatosis through autophagy activation. These include pioglitazone (the PPAR-γ agonist) [79] and gemigliptin (the DPP-4 inhibitor) [80] from the category of antidiabetic agents, irbesartan [81] among antihypertensive drugs, the nonsteroidal anti-inflammatory drugs celecoxib [82] and valdecoxib [83] and the FXR agonist obeticholic acid [84]. These findings are summarised in Table 2.

### 4.3. Plant-Derived Compounds

In recent years, plant extracts have been increasingly shown to improve NAFLD by modulating autophagy. Given the wide variety of compounds, we have organised the relevant findings based on autophagy-related signalling pathways and their targeted mechanisms involved in NAFLD pathogenesis (Table 3).

#### 4.3.1. Autophagy Involved in the AMPK Signalling Pathway

AMPK is a key regulator of energy metabolism and plays an essential role in hepatic lipid homeostasis. Activation of AMPK and its associated signalling pathways represents an important mechanism by which many bioactive compounds modulate autophagy [88]. Several plant-derived compounds, including icariin [89], Toona sinensis bark and fruit extracts [90], naringenin [91] and psoralen [92], have been shown to directly promote AMPK phosphorylation, thereby inducing autophagy and reducing hepatic lipid accumulation in models of NAFLD. Schisandrin B, a lignan compound isolated from Schisandra chinensis, activates autophagy via the AMPK and mTOR signalling pathway, suppresses hepatic steatosis, and promotes fatty acid oxidation [93]. Similarly, pterostilbene, extracted from Pterocarpus species, has been shown to activate autophagy by upregulating Nrf2 expression and promoting the AMPK and mTOR pathway, thereby alleviating oxidative stress associated with lipid overload in hepatocytes and enhancing fatty acid catabolism [94]. In addition, several traditional Chinese medicines or plant extracts have been confirmed through in vivo and/or in vitro experiments to ameliorate NAFLD via similar pathways. These include mangiferin [95], atractyloside [96], sweroside [97], thymiquinone [98], and red pepper seeds [99]. Sirt1 also participates in the regulation of hepatic lipid metabolism by inducing autophagy, and it is known to act cooperatively with AMPK. Several compounds, such as ginsenoside Rb2 [100], sodium isosteviol [101], and apple polyphenol extract [102], have been reported to restore hepatic autophagy and improve lipid metabolism in NAFLD through the SIRT1 and AMPK signalling pathway. Furthermore, catalpol [103] and aurantio-obtusin [104] have been shown to alleviate hepatic steatosis by activating AMPK and TFEB-dependent autophagy.

#### 4.3.2. Autophagy Involved in the TFEB Signalling Pathway

Defective fusion between autophagosomes and lysosomes impairs autophagic flux, leading to intracellular lipid accumulation and contributing to the development of NAFLD. TFEB is considered a master regulator of autophagy and lysosomal biogenesis. Under stress conditions, TFEB translocates from the cytoplasm and lysosomal surface into the nucleus, where it promotes lysosomal biogenesis, enhances autophagy, and facilitates mitochondrial fatty acid degradation. Phillyrin, a lignan extracted from Forsythia suspensa, has been shown to restore hepatic lipophagy and reduce lipid accumulation and inflammation by stimulating endoplasmic reticulum calcium release in hepatocytes, thereby activating calcineurin and regulating TFEB dephosphorylation and nuclear translocation [105]. A lead compound IA, isolated from Paeonia lactiflora, has been reported to alleviate high-fat diet (HFD)-induced liver injury by activating farnesoid X receptor (FXR) and promoting lipid degradation through TFEB-mediated autophagy induction [106]. Similarly, compounds such as ajugol [107], polydatin [108], nuciferine [109], and formononetin [110] have also been found to restore autophagic flux and alleviate NAFLD by enhancing TFEB-mediated autophagy–lysosome pathways and lipid-specific autophagy. Current research has primarily focused on animal models and cellular experiments, with no direct reports of clinical trials specifically investigating autophagy pathways such as AMPK/mTOR and TFEB in human NAFLD. Future human clinical trials are needed to validate the translational effects of these pathways in NAFLD treatment, particularly regarding the clinical safety and efficacy of TFEB activators or AMPK/mTOR modulators.

#### 4.3.3. Autophagy Involved in Oxidative Stress and Endoplasmic Reticulum Stress (ERS) Pathways

ERS and oxidative stress are major drivers of the onset and progression of NAFLD. Excessive ERS and oxidative stress generate reactive oxygen species that damage mitochondria, and because autophagosome formation usually begins on mitochondrial or endoplasmic reticulum membranes, inappropriate ERS and oxidative stress can impair autophagy to some extent [111]. Quercetin, a flavonoid polyphenol with antioxidant and immunomodulatory activities, reduces hepatic triglyceride content through the IRE1α/XBP1s pathway, increases very-low-density lipoprotein assembly and lipophagy, and thereby mitigates high-fat diet-induced NAFLD [112]. Scutellarin suppresses the IRE1α/XBP1 branch, up-regulates Foxo1-mediated autophagy, and the resulting autophagy activation relieves ERS and ultimately down-regulates SREBP-1c-dependent lipogenesis [113]. Recent evidence indicates that aescin promotes hepatic autophagy by activating the Keap1/Nrf2 antioxidant pathway, improving lipid accumulation in NAFLD [114]. Zhang and colleagues reported that physalin B extracted from Physalis species increases the autophagy markers p62 and LC3 II/I while activating the p62/Keap1/Nrf2 antioxidant pathway, which alleviates hepatic oxidative stress and improves nonalcoholic steatohepatitis [115]. These findings suggest that autophagy, oxidative stress, and ERS regulate one another and together play critical roles in the progression of NAFLD.

#### 4.3.4. Autophagy Involved in Inflammatory Pathways

Inflammation is a hallmark feature of NASH. Lipid overload in hepatocytes causes lipotoxicity, which promotes the release of damage-associated molecular patterns (DAMPs). These DAMPs can bind to pattern recognition receptors (PRRs), thereby activating hepatic immune responses involving resident Kupffer cells and other inflammatory cells, leading to a cascade of inflammatory signalling events [116]. Previous studies have shown that defective autophagy exacerbates hepatic inflammation in NAFLD, whereas activation of autophagy alleviates hepatic steatosis and inflammation [117,118]. Scoparone, a natural bioactive compound isolated from Fritillaria, has been reported to enhance macrophage autophagy and suppress inflammation by modulating the ROS/P38/Nrf2 axis and the PI3K/AKT/mTOR signaling pathway in macrophages [119]. Similarly, glycyrrhetinic acid, extracted from licorice root, alleviates impaired autophagic flux and excessive hepatocyte apoptosis by regulating the STAT3 and HIF-1α pathway in macrophages, resulting in reduced production of inflammatory cytokines [120]. In addition, other plant-derived compounds such as phloretin [121], resveratrol [118], and magnolol [122] have been shown to activate hepatic autophagy, reduce inflammation, and attenuate liver injury associated with NAFLD.

#### 4.3.5. Mitophagy

Mitophagy, the selective degradation of damaged or dysfunctional mitochondria via the autophagic pathway, has emerged as a key cellular process for maintaining mitochondrial quality control [4]. Several compounds have been shown to improve NAFLD by enhancing mitophagy, offering new therapeutic possibilities [123]. Mechanistic studies have revealed that cyanidin-3-O-glucoside (C3G) improves hepatic steatosis and glucose metabolism by upregulating the expression and mitochondrial localisation of PINK1 and Parkin, thereby promoting PINK1-mediated mitophagy [124]. Akebia saponin D (ASD), the most abundant component in the rhizome of Dipsacus asper, has been reported to reduce hepatic lipid accumulation by targeting BNip3-mediated mitophagy [125]. Two independent studies on quercetin have shown that it attenuates liver injury, histopathological changes, and lipid metabolism disturbances in NAFLD by activating mitophagy through both AMPK-dependent and frataxin-regulated PINK1/Parkin-dependent pathways [126,127]. These findings suggest that enhancing selective mitophagy may represent a promising strategy for the treatment of NAFLD.

**Table 3:** Herbal medicines or plant extracts associated with autophagy induction for NAFLD treatment.

Items	Medicines/Plant Extracts	Sources/Properties	NAFLD Models	Mechanisms for Improving NAFLD	Years	Ref
AMPK-related	Icaritin	*Herba Epimedii*	Huh-7/L02 cells + sodium oleate	Increasing energy expenditure and regulating autophagy by	2021	[89]
autophagy				activating the AMPK pathway		
	Bark and fruit extracts	-	HepG2 cells + FFAs	Activating the AMPK pathway and upregulating	2019	[90]
	of *Toona sinensis*			the autophagic flux		
	Naringenin	Fruits, vegetables and nuts	Sprague-Dawley male rats fed HFD,	Enhancing energy expenditure and regulating autophagy	2021	[91]
			Huh-7/L02 cells + sodium oleate	via AMPK		
	Psoralen	Buguzhi	L02 cells + sodium oleate	Alleviating IR and promoting autophagy via AMPK	2022	[92]
	Schisandrin B	Schisandra chinensis	HepG2 cells/MPHs + FFAs	Activation of autophagy through the AMPK/mTOR pathway	2022	[93]
	Pterostilbene	Pterocarpus, blueberry	C57BL/6 male mice injected with tyloxapol,	Activation of the AMPK/mTOR pathway and autophagy	2023	[94]
		and grape plants	HepG2 + FFAs	by promoting Nrf2		
	Mangiferin	Mango	Kunming male mice fed HFD	Regulation of autophagy through the AMPK/mTOR pathway	2017	[95]
	Atractyloside	A diterpenoid glycoside	ICR male mice fed HFD	Activation of autophagy via the ANT-AMPK-mTORC1 pathway	2021	[96]
	Sweroside	Alfalfa buds	C57BL/6J male mice fed HFD, MPHs + PA	Activating AMPK/mTOR-mediated autophagy	2023	[97]
	Thymoquinone	Seeds of *Nigella sativa*	C57BL/6N mice fed HFD, HepG2 cells + FFAs	Inducing autophagy via AMPK/mTOR/ULK1-dependent	2023	[98]
				signaling pathway		
	Red pepper seed extract	-	C57BL/6 male mice fed HFD, HepG2 cells + OA	Downregulation of hepatic lipids via AMPK/mTOR pathway	2022	[99]
	Ginsenoside Rb2	Panax ginseng	ob/ob male mice fed NCD, HepG2 cells/MPHs + OA	Restoring autophagy via induction of sirt1	2017	[100]
				and activation of AMPK		
	Isosteviol sodium	*Stevia rebaudiana*	Sprague-Dawley male rats fed HFD, LO2 cells + FFAs	Initiating autophagy via the Sirt1/AMPK pathway	2022	[101]
	Apple polyphenol extract	-	HepG2 cells + FFAs	Activation of autophagy mediated by SIRT1/AMPK signalling	2021	[102]
	Catalpol	*Rehmannia*	C57BL/6 male mice fed HFD, ob/ob male	Through AMPK/TFEB-dependent autophagy	2019	[103]
			mice fed NCD, HepG2 cells + PA			
	Aurantio-obtusin	*Cassia* semen	C57BL/6J male mice fed HFSW, MPHs + FFAs	Through AMPK/autophagy- and AMPK/TFEB-mediated	2022	[104]
				suppression of lipid accumulation		
TFEB-related	Phillygenin	*Forsythia suspense*	C57BL/6J male mice fed HFD,AML-12/MPHs + PA	Through regulating the Ca2+-calcineurin-TFEB axis to	2022	[105]
autophagy				restore lipophagy		
	Isopropylidenyl	Chi-Shao	C57BL/6N male mice fed CDAHFD, Sprague-	Through FXR activation and TFEB-mediated autophagy	2022	[106]
	anemosapogenin		Dawley rats induced BDL, LX-2 cells+			
			TGF-β1, Huh7 cells + OA			
	Ajugol	*Rehmannia glutinosa*	C57BL/6 male mice fed HFD, AML-12 cells + PA	Through the TFEB-mediated autophagy-lysosomal pathway and lipophagy	2021	[107]
	Polydatin	A precursor of resveratrol	db/db mice fed MCD, LO2 cells + PA	Restoring lysosomal function and autophagic flux through TFEB	2019	[108]
	Nuciferine	Lotus leaf	C57BL/6N male mice fed HFD, MPHs/AML12 cells + PA	Activating TFEB-mediated autophagy-lysosomal pathway	2022	[109]
	Formononetin	A natural isoflavone	C57BL/6J mice fed HFD, HepG2 cells/MPHs + FFAs	Through TFEB-mediated lysosome biogenesis and lipophagy	2019	[110]
Oxidative stress	Quercetin	Flavonoid polyphenols	Sprague-Dawley male rats fed HFD, HepG2 cells + FFA	Promoting VLDL assembly and lipophagy via the IRE1a/XBP1s pathway	2018	[112]
and ERS-related	Scutellarin	*Erigeron breviscapus*	C57BL/6 male mice fed HFD, HepG2 cells/MPHs + PA	Enhancing autophagy and inhibiting ERS via the IRE1α/XBP1 pathway	2022	[113]
autophagy	Aescin	Aesculus chinensis Bunge	C57BL/6male mice fed HFD,HepG2 cells + FFAs	Activation of antioxidant and autophagy via the Keap1-Nrf2 pathway	2023	[114]
	Physalin B	*Physalis* species	C57BL/6J mice fed MCD,LO2 cells + FFA	Stimulating autophagy and P62-KEAP1-NRF2 antioxidative signalling	2021	[115]
Inflammation-	Scoparone	*Artemisia capillaris*	C57BL/6J mice fed MCD,AML-12 cells + PA,	Inhibiting ROS/P38/Nrf2 axis and PI3K/AKT/mTOR pathway	2020	[119]
related			RAW264.7 cells + LPS	and enhancing autophagic flux in macrophages		
autophagy	Glycyrrhetinic acid	*Glycyrrhiza uralensis*	C57BL/6 male mice fed HFHFr, RAW264.7 cells + PA	Modulating macrophage STAT3-HIF-1α pathway and	2022	[120]
				ameliorating impaired autophagic flux		
	Phloretin	Apple fruits	C57BL/6Jmale mice fed WD, Huh7 cells + FFA	Mitigating oxidative damage, inflammation, and fibrotic responses	2022	[121]
				by restoring autophagic fluxes		
	Resveratrol	A polyphenol	C57BL/6male mice fed MCD, AML12 cells + MCD medium	Lessening hepatic inflammation by modulating autophagy	2015	[118]
	Magnolol	*Magnolia officinalis*	Wistar male rats injected with tyloxapol,	Inhibition of NLRP3 inflammasome activation	2020	[122]
			HepG2 cells + PA	by restoration of autophagy		
Mitophagy	cyanidin-3-O-	An anthocyanin in	C57BL/6 mice fed HFD, AML-12/HepG2 cells + PA	Promoting PINK1-mediated mitophagy	2020	[124]
	glucoside	flavonoids				
	Akebia Saponin D	Dipsacus asper Wall	BRL cells + OA	Through BNip3-mediated mitophagy	2018	[125]
	Quercetin	A flavonoid	C57BL/6Jmale mice fed HFD, HepG2 cells + OA/PA	Enhancing frataxin-mediated PINK1/Parkin-dependent mitophagy	2018	[127]
			C57BL/6J male mice fed MCD, HepG2 cells + OA	Through AMPK-mediated hepatic mitophagy	2023	[126]

### 4.4. Others

#### 4.4.1. Hormones

Thyroid hormones (THs) play an essential role in organ development, cellular differentiation, and the regulation of protein, carbohydrate, and lipid metabolism. Increasing evidence has indicated that reduced thyroid hormone activity is associated with an elevated risk of NAFLD [128]. However, TH supplementation may exert protective effects against NAFLD, partly through the induction of hepatic autophagy [129]. Recent studies have demonstrated that administration of T3 or T4 in NASH mouse models restores hepatic autophagy and mitochondrial biogenesis, thereby enhancing fatty acid β-oxidation and reducing lipotoxicity, oxidative stress, inflammation, and fibrosis [130]. Another study investigating the metabolic effects of THs on the liver supports their therapeutic potential in improving liver homeostasis [131].

In addition, melatonin has also shown beneficial effects in improving NAFLD to some extent [132]. In a cadmium-induced NAFLD model, melatonin attenuates mitochondrial damage, oxidative stress, and hepatic lipid accumulation by restoring PPARα expression and autophagic flux [133]. Furthermore, melatonin supplementation has been shown to improve mitochondrial and liver function in NAFLD by restoring mitophagy through inhibition of the NR4A1/DNA-PKcs/p53 signalling pathway [134].

#### 4.4.2. Nanoparticles

Nanoparticles (NPs) are particulate materials typically ranging in size from approximately 50 to 200 nanometers. Due to their favourable characteristics, including high drug-loading capacity, variable shapes and sizes, and stable ligand binding, NPs have emerged as promising tools for targeted therapeutic delivery and modulation of cellular processes. They have shown great potential in medical applications [135]. In a study on acid-activated acidifying NPs for targeted therapy of NAFLD in mice, these NPs were able to re-acidify lysosomes, restore autophagy and mitochondrial function, and reverse fasting hyperglycemia and hepatic steatosis in the treated animals [136]. Another investigation demonstrated that lycopene-loaded nanoliposomes significantly attenuated oxidative stress, inflammation, and apoptosis in liver tissue while inducing autophagy, thereby exerting therapeutic effects against NAFLD [137]. Additionally, researchers have developed nifedipine-loaded nanoparticles, which not only enhanced autophagic clearance in hepatocytes but also improved insulin resistance and glucose tolerance in obese mice and mitigated metabolic disturbances associated with NAFLD [138]. More importantly, nanoparticles loaded with the autophagy-inducing peptide Tat-Beclin (T-B) have been engineered. Compared to the soluble peptide alone, the nanoparticle formulation induced more sustained and potent autophagic activity. Both T-B and NP-T-B effectively reduced lipid accumulation in NAFLD cellular models [139]. These findings suggest that nanoparticles may hold broad therapeutic potential in the treatment of NAFLD through autophagy modulation and targeted metabolic regulation.

#### 4.4.3. Gut Microbiota

The gut microbiota, consisting of bacteria, archaea, viruses, and fungi residing in the human gastrointestinal tract, plays a critical role in host physiology. Dysbiosis of the gut microbiota is not only a characteristic feature of NAFLD but also contributes significantly to its pathogenesis [140]. Recent studies have demonstrated that supplementation with selenium-enriched probiotics alleviates liver dysfunction and hepatic steatosis in NAFLD rats by activating autophagy through the AMPK and SIRT1 signalling pathway [141]. Furthermore, it has been reported that Urolithin A, a gut microbiota-derived metabolite, promotes hepatic lipophagy via the AMPK and ULK1 pathway both in vitro and in vivo. This compound suppresses lipogenesis, enhances fatty acid β-oxidation, and reduces lipid overaccumulation in the liver, thereby improving NAFLD [142]. In addition, Lactobacillus bifidus SF has been shown to reduce inflammation and hepatic lipid accumulation by mitigating oxidative stress and regulating autophagy, which together contribute to the improvement of NAFLD [143].

#### 4.4.4. Vitamins

Vitamins are small organic molecules that play essential roles in supporting growth and development, maintaining physiological functions, and regulating metabolic processes [144]. Clinical trials have provided evidence that vitamin supplementation may offer therapeutic benefits in the treatment of NAFLD, and the underlying mechanisms are increasingly thought to involve autophagy induction [145,146]. Vitamin D3, a steroid hormone, exerts its biological activity primarily through binding to the vitamin D receptor (VDR) in the form of 1,25-dihydroxyvitamin D3 [1,25(OH)_2_D_3_] [144]. Li and colleagues [147] reported that intraperitoneal injection of 1,25(OH)_2_D_3_ for 4 weeks in high-fat diet-fed mice alleviated hepatic lipid accumulation, possibly through the upregulation of ATG16L1 to promote autophagy. Lim and colleagues [148] further explored the autophagy-related mechanisms of vitamin D3 in a NAFLD model with type 2 diabetes mellitus. Their findings showed that under high-glucose conditions, vitamin D3 improved hepatic lipid metabolism by activating autophagy through regulation of the AMPK/Akt–mTOR pathway. In addition, another study demonstrated that supplementation with nicotinamide, the amide form of vitamin B3, protected hepatocytes from palmitic acid-induced lipotoxicity via SIRT1-dependent autophagy activation [149]. These findings suggest that the induction of autophagy may represent a key mechanism through which vitamins exert hepatoprotective effects. Further details are summarised in Table 4.

## 5. Conclusions and Outlook

NAFLD is a common chronic liver disease caused by multiple etiologies. Histologically, it can evolve from simple hepatic steatosis eventually to hepatocellular carcinoma. Hence, it is particularly important to explore the pathogenesis and treatment of NAFLD. Autophagy, a process by which eukaryotic cells clean up damaged or excess intracellular components under stress, has a crucial role in maintaining metabolic homeostasis in the liver. A growing number of studies have pointed out that impaired autophagy exists in NAFLD, and impaired autophagy can be accompanied by inflammation, ERS, and apoptosis, which further aggravate NAFLD and form a vicious circle. Thus, restoration of autophagy may become an important line of thought in therapy for NAFLD. In the present study, we summarise the latest literature on the treatment of NAFLD from the perspective of restoring or inducing autophagy in terms of diet and exercise, drugs (modern pharmacological therapy and plant-derived compounds), and other measures (hormones, nanoparticles, gut microbes, and vitamins). The potential molecular targets of NAFLD related to autophagy are also briefly described, hoping to provide some reference for future research on autophagy and NAFLD treatment. However, since most of the above findings are derived from cell or animal trials and the elaboration of the degree of autophagy induction is vague, there is still an urgent need for a large amount of clinical evidence to validate their efficacy and safety in the future.

## Figures and Tables

**Table 1:** Diet and exercise regimens associated with autophagy induction in treating NAFLD.

Items	NAFLD Models	Mechanisms for Improving NAFLD	Years	Ref
IF	C57BL/6J male mice fed HFD	Activation of MIF/AMPK pathway and attenuation of lipotoxicity	2023	[27]
ADF	Sprague Dawley male rats fed HFF	Modulation of adipogenesis, autophagy, apoptosis and inflammation	2021	[28]
ADF	C57BL/6J male mice fed HFD	Through activation of the AMPK/ULK1 pathway and inhibition of mTOR phosphorylation	2022	[29]
Increasing the proportion	C57BL/6 male mice fed HFD	Restoring autophagy inhibited by rubicon	2017	[30]
of medium-chain fatty acids	HepG2 cells + FA			
Corn peptides	Sprague Dawley male rats fed HFD	Through PINK1/Parkin-mediated mitophagy	2023	[31]
	HepG2 cells + FFA			
γ-Linolenic acid	AML-12 cells + PA	Balancing autophagy and apoptosis through the LKB1-AMPK-mTOR pathway	2021	[32]
Voluntary wheel running	C57BL/6J mice fed WD	Enhancing basal autophagy in the liver	2017	[37]
Endurance exercise	C57BL/6J female mice fed HFD/HF	Prevention of autophagy deficiency	2022	[38]
Treadmill exercise	C57BL/6 male mice fed HFD	Regulating the biogenesis and autophagy of lipid droplets	2022	[41]
Voluntary physical activity	Sprague Dawley male rats fed HFD	Promotion of autophagy/mitochondrial autophagy and mitochondrial fusion	2016	[42]
and endurance training				
Swimming	C57BL/6J male mice fed HFD	Restoration of autophagy-lysosomal machinery by reducing FABP1	2019	[44]
Treadmill exercise	C57Bl/6N male mice fed CD-HFD	Through the AMPK/mTOR pathway to promote hepatic autophagy	2020	[45]
Swim training	C57BL/6J male mice fed HFD	Through the AMPK/SIRT1 pathway and lipophagy	2021	[46]
Treadmill running+	Sprague Dawley male rats fed HFD	Activation of AMPK/ULK1 and inhibition of Akt/mTOR/ULK1 pathway to enhance lipophagy	2020	[48]
dietary intervention				
Treadmill running + DHA	C57BL/6J female mice fed HFD	Inhibition of inflammation and autophagy marker alterations and promotion of FAO	2021	[47]

**Table 2:** Modern pharmacological therapy related to autophagy induction in treating NAFLD.

Items	Western Medicines	NAFLD Models	Mechanisms for Improving NAFLD	Years	Ref
SGLT-2i	Empagliflozin	C57BL/6J male mice fed HFD	Negative regulation of the IL-17/IL-23 axis through	2021	[56]
			AMPK/mTOR autophagy pathway		
		ApoE^−/−^ male mice fed HFD	Activation of autophagy and reduction of ERS and apoptosis	2021	[85]
		C57BL/6J male mice fed AMLN, Liver samples from subjects	Activation of the AMPK-TFEB pathway by reducing	2023	[57]
		with NASH, HepG2/MIHA cells + FFA	O-GlcNAcylation and promotion of autophagic flux		
	Canagliflozin	C57BL/6J male mice fed HFD, AML-12 cells + LM	Regulation of lipid metabolism and inhibition of	2022	[58]
			Inflammation by inducing autophagy		
	Dapagliflozin	ZDF male rats fed HFD, HepG2/LO2 cells + PA	Through the AMPK/mTOR pathway to restore autophagy	2021	[59]
GLP1-RA	Liraglutide	C57BL/6J male mice fed HFD	Through the SIRT1/SIRT3-FOXO3a pathway to enhance autophagy	2016	[63]
			and reinforce mitochondrial structure		
		HepG2 cells + PA + LPS	Through mitophagy to inhibit the NLRP3 inflammasome	2019	[64]
			and hepatocyte pyroptosis		
		C57BL/6J male mice fed HFD, LO2 cells + FFA	Induction of autophagy through the AMPK/mTOR pathway	2016	[86]
		Rora LKO C57BL/6Jmice fed HFD,	Through the RORα-mediated autophagy pathway	2023	[87]
		Rora LKO AML-12 cells + PA			
	Exenatide	C57BL/6J male mice fed HFD	Through the mitophagy pathway to inhibit the NLRP3 inflammasome	2018	[65]
		HepG2 cells + PA/OA	Enhancing the autophagy-lysosomal pathway	2022	[66]
		LO2/HepG2 cells + PA	Activation of the AKT/mTOR pathway to promote autophagy	2021	[67]
Biguanides	Metformin	C57BL/6J male mice fed MCD, AML-12 + MCD medium	Reducing hepatic inflammation through STAT3-mediated autophagy	2019	[69]
		Ob/ob mice fed NCD, MHPs/HepG2	Restoration of SIRT1-mediated autophagy via	2015	[70]
		cells + OA + high glucose	AMPK-independent pathway		
		Ob/ob mice fed NCD, HepG2 cells + OA + high glucose	Restoration of parkin-mediated mitophagy	2016	[71]
		C57BL/6J male mice fed HFD	Through TFEB-dependent autophagy	2021	[72]
		Ttp^+/+^/Ttp^−/−^ mice fed MCD, AML12 + MCD medium	Promoting lipophagy via mTORC1 inhibition and increased	2023	[73]
			nuclear TFEB		
Lipid-modifying	Fenofibrate	TFEB knockdown mice fed HFD	Through the upregulation of TFEB-mediated lipophagy	2021	[75]
drugs					
		C57BL/6J male mice fed HFD	Through TFEB-autophagy axis	2023	[76]
	Ezetimibe	NAFLD patients, C57BL/6J male mice fed MCD	Through p62-dependent Nrf2 activation	2016	[78]
		Obese and diabetic male rats, Huh7cells + PA	Promoting autophagy gene expression and increasing autophagic flux	2015	[77]
Others	Pioglitazone	C57BL/6J male mice fed HFD, AML12cells + PA	Enhancing cytosolic lipolysis, β-oxidation and autophagy	2017	[79]
			in a PPARα and PPARγ dependent manner		
	Gemigliptin	NAFLD patients, C57BL/6NCrj male mice fed	Through AMPK-independent, ULK1-mediated effects on autophagy	2023	[80]
		MCD, HepG2 cells + MCD medium			
	Irbesartan	LO2/AML-12 cells + FFA	Induction of autophagy through the PKC/AMPK/ULK1 axis	2019	[81]
	Celecoxib	Sprague Dawly male rats fed HFD, LO2 cells + PA	Restoration of autophagic flux by downregulating COX-2	2018	[82]
	Valdecoxib	C57BL/6J male mice fed HFD, MPHs + PA	Inhibition of ERS through AMPK/SIRT6 autophagy pathway	2022	[83]
	Obeticholic	Swiss albino male mice fed HFD and dextran sulfate sodium	Through autophagy induction via interfering with the TLR4/TGF-β1	2022	[84]
	acid		pathway to protect intestinal integrity in NASH		

**Table 4:** Alternative approaches to treating NAFLD related to autophagy induction.

Items	Others	NAFLD Models	Mechanisms for Improving NAFLD	Years	Ref
Hormones	T3/T4	C57BL/6J male mice fed WDF,	Restoration of autophagy and mitochondrial biogenesis	2022	[130]
		HepG2-TRβ cells + PA/LPS	and promotion of fatty acid β-oxidation		
	T2/T3	Wistar male mice fed HFD	Strong induction of autophagy and intrahepatic	2017	[131]
			acylcarnitine flux		
	Melatonin	Shaoxing male ducks fed Cd	Restoring the expression of PPAR-α and autophagy flux	2023	[133]
		AML-12 cells + cadmium chloride			
		C57BL/6J male mice fed HFD	Restoration of autophagy flux by regulating miR-34a-5p/Sirt1 axis	2019	[150]
		C57BL/6 mice fed HFD	Restoration of mitophagy by blocking NR4A1/DNA-PKcs/p53 pathway	2018	[135]
NPs	acNPs	C57BL/6J male mice fed HFD,	Restoration of autophagy and mitochondrial	2023	[136]
		HepG2 cells + PA	function by lysosomal acidification		
	Lip-Lyco	Sprague Dawley male rats fed HFD	Exhibiting antioxidant, anti-inflammatory, hypoglycemic,	2023	[137]
			antiapoptotic, and autophagy-inducing		
	NFD-NPs	C57BL/6 male mice fed HFD,	Enhancing autophagic clearance through Ca2+/CaMKII phosphorylation	2019	[138]
		HepG2 cells + PA			
	NP T-B	ob/ob male mice fed NCD,	Inducing autophagy with a long-lasting and enhanced effect	2022	[139]
		HeLa cells + FFAs			
Intestinal microbiota	SP	Sprague Dawley male rats fed HFHFr	Modulation of autophagy through AMPK/SIRT-1 pathway	2023	[141]
	UroA	C57BL/6 female mice fed HFrD,	Facilitating hepatic lipophagy through the AMPK/ULK1 pathway	2023	[142]
		HepG2 cells/MPHs + fructose			
	B. lactis SF	C57BL/6N male mice fed HFD	Reduction of OS and autophagy	2023	[143]
Vitamins	1,25(OH)_2_ D_3_	C57BL/6 male mice fed HFD,	Induction of autophagy by upregulation of ATG16L1	2017	[147]
		HepG2 cells + FFA			
	Vitamin D3	C57BL/6J male mice fed HFD	Activating autophagy regulatory AMPK/Akt-mTOR signalling	2021	[148]
	Vitamin B3	HepG2 cells + PA	Through SIRT1-dependent autophagy	2017	[149]

## Data Availability

The data generated by this study will be made available upon request.

## Supplementary Materials

The following supporting information can be downloaded at: https://www.mdpi.com/article/10.3390/biology14080989/s1, Table S1: A comprehensive clinical comparison between SGLT-2i and GLP-1RA. Refs. [151,152,153,154,155,156,157,158,159,160,161,162].
