# Supplementary material for: Induction of Autophagy as a Therapeutic Breakthrough for NAFLD: Current Evidence and Perspectives
Source: Biology (Basel). 2025 Aug 4;14(8):989. doi: 10.3390/biology14080989 (PMC12383869; doi:10.3390/biology14080989)
Supplement: Supplementary file 1 [file biology-14-00989-s001.zip › biology-3787619-supplementary.pdf]

Table S1. A comprehensive clinical comparison between SGLT-2i and GLP-1RA

Parameter	SGLT2 Inhibitors	GLP-1 Receptor Agonists	Clinical Implications	Ref
Glycemic Efficacy	HbA1c reduction: 0.5-1.0%	HbA1c reduction:1.0-1.8%	GLP-1RAs show superior glucose-lowering effects	[151,152]
Weight Loss	Average 2-3 kg	Average 5-10 kg	GLP-1RAs preferred for obese patients	[153,154]
Cardiovascular Benefits	Clear benefits in heart failure (HF) and chronic kidney disease (CKD)	Atherosclerotic cardiovascular events (ASCVD) and metabolic improvement	SGLT2i for HF, GLP-1RA for ASCVD	[155,156]
Renoprotection	Potent delay in eGFR decline, reduction in end-stage renal disease and renal mortality	Mildly reduce proteinuria and help delay eGFR decline	SGLT2i superior for CKD	[156]
Hepatic Benefits	Mildly reduce liver fat and improve liver function parameters (primarily in NAFLD)	Significantly reduce liver fat and alleviate NASH inflammation and fibrosis (effective in both NAFLD and NASH)	GLP-1RAs more effective for NAFLD and NASH	[157]
Common Side Effects	Genitourinary tract infections and blood volume depletion	Gastrointestinal reactions (nausea/vomiting) and injection site reactions	Selection based on patient tolerance	[158,159]
Administration	Oral (once daily)	Subcutaneous injection<br>(daily or weekly)	Better adherence with SGLT2i	[160]
Ideal Patient Profile	T2DM with HF/CKD	T2DM with obesity/ASCVD	Individualize based on comorbidities	[155,156]
Contraindications	Severe renal impairment and recurrent genital infections	History of medullary thyroid carcinoma<br>and pancreatitis	Requires careful screening	[158,161]
Cost	Lower cost	Higher cost	SGLT2i more cost-effective	[162]

Reference:

151. Palecek, E.J.; Kimzey, M.M.; Zhang, J.; Marsden, J.; Bays, C.; Moran, W.P.; Mauldin, P.D.; Schreiner, A.D. Glucagon-like peptide-1 receptor agonist therapy effects on glycemic control and weight in a primary care clinic population. J Investig Med. 2024, 72, 911-919.
152. Suzuki, A.; Hayashi, A.; Oda, S.; Fujishima, R.; Shimizu, N.; Matoba, K.; Taguchi, T.; Toki, T.; Miyatsuka, T. Prolonged impacts of sodium glucose cotransporter-2 inhibitors on metabolic dysfunction-associated steatotic liver disease in type 2 diabetes: a retrospective analysis through magnetic resonance imaging. Endocr J. 2024, 71, 767-775.
153. Kumar, N.; D'Alessio, D.A. Slow and Steady Wins the Race: 25 Years Developing the GLP-1 Receptor as an Effective Target for Weight Loss. J Clin Endocrinol Metab. 2022, 107, 2148-2153.
154. Pereira, M.J.; Eriksson, J.W. Emerging Role of SGLT-2 Inhibitors for the Treatment of Obesity. Drugs. 2019, 79, 219-230.

155. van Ruiten, C.C.; Hesp, A.C.; van Raalte, D.H. Sodium glucose cotransporter-2 inhibitors protect the cardiorenal axis: Update on recent mechanistic insights related to kidney physiology. Eur J Intern Med. 2022, 100, 13-20.
156. Gómez-Huelgas, R.; Sanz-Cánovas, J.; Cobos-Palacios, L.; López-Sampalo, A.; Pérez-Belmonte, L.M. Glucagon-like peptide-1 receptor agonists and sodium-glucose cotransporter 2 inhibitors for cardiovascular and renal protection: A treatment approach far beyond their glucose-lowering effect. Eur J Intern Med. 2022, 96, 26-33.
157. Xu, R.; Liu, B.; Zhou, X. Comparison of Glucagon-Like Peptide-1 Receptor Agonists and Sodium-Glucose Cotransporter Protein-2 Inhibitors on Treating Metabolic Dysfunction-Associated Steatotic Liver Disease or Metabolic Dysfunction-Associated Steatohepatitis: Systematic Review and Network Meta-Analysis of Randomised Controlled Trials. Endocr Pract. 2025, 31, 521-535.
158. Wiegley, N.; So, P.N. Sodium-Glucose Cotransporter 2 Inhibitors and Urinary Tract Infection: Is There Room for Real Concern? Kidney360. 2022, 3, 1991-1993.
159. Gorgojo-Martínez, J.J.; Mezquita-Raya, P.; Carretero-Gómez, J.; Castro, A.; Cebrián-Cuenca, A.; de Torres-Sánchez, A.; García-de-Lucas, M.D.; Núñez, J.; Obaya, J.C.; Soler, M.J.; Górriz, J.L.; Rubio-Herrera, M. Clinical Recommendations to Manage Gastrointestinal Adverse Events in Patients Treated with Glp-1 Receptor Agonists: A Multidisciplinary Expert Consensus. J Clin Med. 2022, 12, 145.
160. D'Marco, L.; Morillo, V.; Gorriz, J.L.; Suarez, M.K.; Nava, M.; Ortega, Á.; Parra, H.; Villasmil, N.; Rojas-Quintero, J.; Bermúdez, V. SGLT2i and GLP-1RA in Cardiometabolic and Renal Diseases: From Glycemic Control to Adipose Tissue Inflammation and Senescence. J Diabetes Res. 2021, 2021, 9032378.
161. Wilbon, S.S.; Kolonin, M.G. GLP1 Receptor Agonists-Effects beyond Obesity and Diabetes. Cells. 2023, 13, 65.
162. Choi, J.G.; Winn, A.N.; Skandari, M.R.; Franco, M.I.; Staab, E.M.; Alexander, J.; Wan, W.; Zhu, M.; Huang, E.S.; Philipson, L.; Laiteerapong, N. First-Line Therapy for Type 2 Diabetes With Sodium-Glucose Cotransporter-2 Inhibitors and Glucagon-Like Peptide-1 Receptor Agonists : A Cost-Effectiveness Study. Ann Intern Med. 2022, 175, 1392-1400.
